# What is the key mediator of the neurovascular coupling response?

**DOI:** 10.1016/j.neubiorev.2018.11.011

**Published:** 2019-01

**Authors:** Patrick S. Hosford, Alexander V. Gourine

**Affiliations:** a Centre for Cardiovascular and Metabolic Neuroscience, Neuroscience, Physiology & Pharmacology, University College London, London, UK; b William Harvey Research Institute, Barts and The London School of Medicine and Dentistry, London, UK

**Keywords:** Brain blood flow, Functional hyperaemia, Neurovascular coupling, Systematic review, Functional MRI

## Abstract

The mechanisms of neurovascular coupling contribute to ensuring brain energy supply is sufficient to meet demand. Despite significant research interest, the mechanisms underlying increases in regional blood flow that follow heightened neuronal activity are not completely understood. This article presents a systematic review and analysis of published data reporting the effects of pharmacological or genetic blockade of all hypothesised signalling pathways of neurovascular coupling. Our primary outcome measure was the percent reduction of the neurovascular response assessed using *in vivo* animal models. Selection criteria were met by 50 primary sources reporting the effects of 79 treatments. Experimental conditions were grouped into categories targeting mechanisms mediated by nitric oxide (NO), prostanoids, purines, potassium, amongst others. Blockade of neuronal NO synthase was found to have the largest effect of inhibiting any individual target, reducing the neurovascular response by 64% (average of 11 studies). Inhibition of multiple targets in combination with nNOS blockade had no further effect. This analysis points to the existence of an unknown signalling mechanism accounting for approximately one third of the neurovascular response.

## Highlights

• Cellular and molecular mechanisms underlying increases in regional blood flow in response to neuronal activity are not fully understood.
• We have compared the effects of 79 *in vivo* and 36 *in vitro* experimental attempts to inhibit the neurovascular response.
• Blockade of neuronal NO synthase (nNOS) has the largest effect of any individual target, reducing the neurovascular response by 64%.
• This points to the existence of an unknown key signalling mechanism which accounts for approximately one third of the neurovascular response.

## 1 Introduction

Constant and optimal nutrient and oxygen supply as well as effective removal of metabolic waste products (*e.g.* CO_2_) are ensured by several mechanisms controlling cerebral blood flow. Control of global cerebral circulation is provided by metabolic factors, cerebral autoregulation, and autonomic mechanisms (Willie et al., 2014). Fine-tuning and continuous adjustment of local cerebral blood flow in accord with the levels of neuronal activity and metabolism are achieved by operation of the neurovascular coupling mechanism. The functional significance of this mechanism arises from 1) the inability of the brain to store significant energy reserves; 2) highly dynamic and regionally heterogeneous metabolism; 3) volume constraints imposed by the cranium; and 4) the need to maximise the efficacy of energy use.

The mechanisms underlying neurovascular coupling have been the subject of intense research interest for the past ∼25 years for two important reasons. Firstly, changes in local brain blood flow which follow changes in neuronal activity provide the physiological basis of blood oxygen level dependent (BOLD) functional MRI (fMRI) imaging (Buxton and Frank, 1997). Regional cerebral blood flow changes in humans can, therefore, be measured non-invasively and used as a surrogate to assess changes in the neuronal activity (Attwell and Iadecola, 2002). Secondly, impaired mechanisms controlling brain blood flow may contribute to cognitive impairment and potentially precipitate the development of dementia/ neurodegenerative disease (Iadecola, 2017). However, despite continued experimental scrutiny by many research groups, the cellular and molecular mechanisms of the neurovascular response are not fully understood and/or remain controversial. Here, using meta-analysis, we evaluated the results of experimental studies that aimed to identify the signalling mechanism(s) of the neurovascular coupling response. We analysed the relative efficacy of experimental treatments targeting all the hypothesised pathways. Our analysis suggests that a substantial proportion of the neurovascular response is mediated by an as yet unknown mechanism.

## 2 Metabolic feedback mechanisms

The physiological significance of the neurovascular response is predicated on the fact that neuronal activity requires copious amounts of energy substrates. Oxygen and glucose are used to generate ATP, which is required to maintain ion gradients and provide energy to fuel the mechanisms of neurotransmitter release/recycling that make neuronal communication and brain information processing possible (Attwell and Laughlin, 2001; Howarth et al., 2012). As levels of neuronal activity are believed to be highly heterogeneous across the brain (which has no significant stores of metabolic substrates), blood flow is redirected to support regions of heightened activity.

Feedback mechanisms are fundamental for the operation of all physiological systems, including those which maintain energy homeostasis. A metabolic feedback hypothesis of brain blood flow regulation was proposed by Roy and Sherrington (1890) who observed that products of cerebral metabolism released during brain asphyxia (hypoxia/hypercapnia) dilate cerebral vasculature. These observations led to the hypothesis that signals increasing local brain blood flow are generated in response to a reduction in supply of metabolic substrates and/or accumulation of metabolic waste products (Kety, 1950).

A reduction in the local concentration of oxygen and/or glucose might, therefore, serve as a suitable negative feedback trigger to initiate the neurovascular response. Brain metabolism is argued to be significantly more sensitive to the decreases of oxygen delivery over that of glucose (Leithner and Royl, 2014). Indeed, experimental studies in anesthetised rats involving raising the arterial concentration of glucose up to ∼18 mmol L^−1^ demonstrated no effect of excess glucose on the neurovascular response (Wolf et al., 1997).

Earlier studies suggested that neurovascular response might be triggered by brain tissue hypoxia associated with the increased neuronal activity (Malonek and Grinvald, 1996; Offenhauser et al., 2005; Thompson et al., 2003). A study conducted in rat brain slices demonstrated that parenchymal oxygen tension determines the polarity of cerebrovascular responses that follow activation of perivascular astrocytes by intracellular Ca^2+^ uncaging (Gordon et al., 2008). More recently astrocytes themselves have been shown to be acutely sensitive to hypoxia (Angelova et al., 2015). Red blood cells have also been proposed to function as oxygen sensors responding with conformational changes to decreases in brain *P*O_2_ during neuronal activation (Wei et al., 2016). Freeman and Li (2016) reported faithful coupling between neuronal activity and local tissue *P*O_2_ decreases upon visual stimulation in the anesthetised cat. However, the magnitude of the neuronal activity-induced transient brain parenchymal *P*O_2_ decreases is small (∼1-5 mmHg) (Freeman and Li, 2016; Parpaleix et al., 2013; Wei et al., 2016) and neurovascular responses are not affected in conditions of normobaric (Wolf et al., 1997) or hyperbaric hyperoxia in rats (Lindauer et al., 2010). In conditions of hyperbaric hyperoxia, enhanced neuronal oxygen consumption would be exceeded by the amount of physically dissolved oxygen. That the neurovascular response is unaffected under hyperbaric hyperoxia would suggest it is unlikely to be triggered by (small) associated decreases in local tissue oxygenation.

Could a build up of metabolic waste products be mediating the neurovascular response? CO_2_, protons and lactate have powerful effects on cerebral vasculature and are generated proportionately with increased brain tissue metabolism.

Astrocytes have been shown to detect local increases in CO_2_ or proton concentrations responding to these stimuli with increases in intracellular [Ca^2+^] (Gourine et al., 2010; Howarth et al., 2017), which may lead to the release of vasoactive signalling molecules. Release of lactate *via* several parallel mechanisms, involving certain membrane channels (Karagiannis et al., 2016; Sotelo-Hitschfeld et al., 2015) is coupled to metabolic signalling pathways (Fernandez-Moncada et al., 2018) and follows enhanced neuronal activity. Yet, the roles of locally produced CO_2_, H^+^ and lactate as potential triggers of the neurovascular response have not been experimentally addressed. There is evidence that brain parenchymal pH does not change or even becomes alkaline due to neuronal activity-induced increases in flow leading to rapid CO_2_ washout and activation of the membrane Ca^2+^-ATPase rapidly removing the excess of extracellular protons (Chen and Chesler, 1992; Makani and Chesler, 2010; Ueki et al., 1988).

Taken together, these lines of evidence argue against the importance of metabolic feedback mechanisms in triggering changes in regional cerebral blood flow which follow changes in the neuronal activity. These mechanisms will not be considered further because of the insufficient number of relevant studies meeting our inclusion criteria for meta-analysis (described in detail below).

## 3 Activity-dependent feed-forward mechanisms

**Fig. 1:**
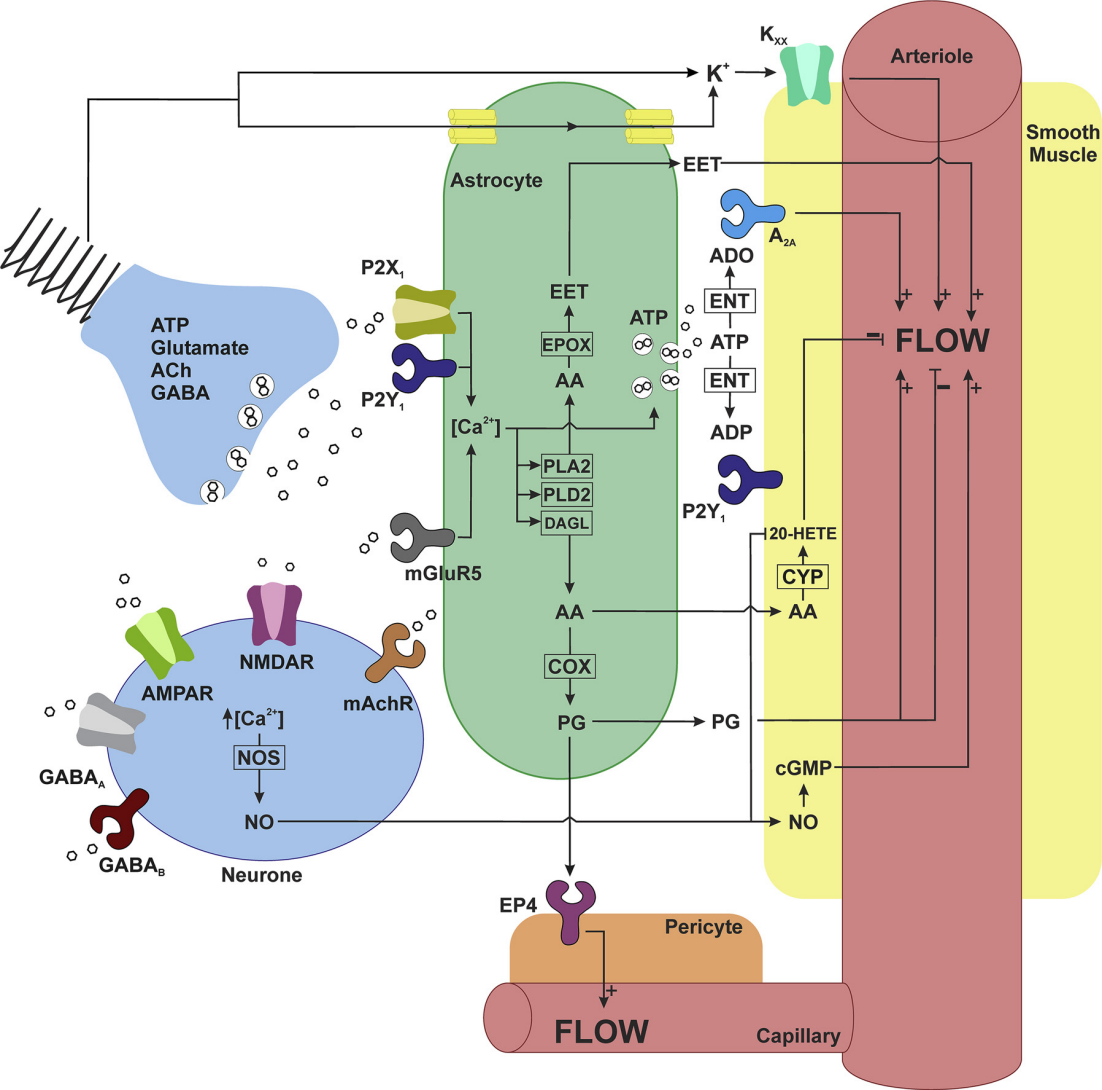
**Hypothesized signalling mechanisms of the neurovascular coupling.** Schematic illustration of all hypothesized pathways mediating the neurovascular response suggested by the results of studies summarized in Figures 2 and 3. All pathways are depicted assuming equal weighting. 20-HETE, 20-Hydroxyeicosatetraenoic acid; A_2A_, adenosine receptor 2A; AA, arachidonic acid; ACh, acetylcholine; ADO, adenosine; ADP, adenosine diphosphate; AMPAR, α-amino-3-hydroxy-5-methyl-4-isoxazolepropionic acid receptor; ATP, adenosine triphosphate; cGMP, cyclic guanosine monophosphate; COX, cyclooxygenase; CYP, cytochrome P450; DAGL, diacylglycerol lipase; EET, epoxyeicosatrienoic acid; ENT, ectonucleotidase; EP4, prostaglandin E_2_ receptor 4; EPOX, cytochrome P450 epoxygenase; GABA_A_, γ-aminobutyric acid (GABA) A receptor; GABA_B_, γ-aminobutyric acid B receptor; K_xx_, potassium channels; mAchR, muscarinic acetylcholine (ACh) receptor; mGluR5, metabotropic glutamate receptor 5; NMDAR, *N*-methyl-d-aspartate receptor; NO, nitric oxide; NOS, nitric oxide synthase; P2X_1_, P2X purinoceptor 1; P2Y_1_, P2Y purinoceptor 1; PG, prostaglandin; PLA2, phospholipase A2; PLD2, phospholipase D2.

An alternative hypothesis to a feed-forward mechanism centres on the idea that neuronal activity-induced changes in the brain neurochemical milieu mediate neurovascular coupling (Attwell et al., 2010). According to this hypothesis, neurotransmitter(s) released during synaptic activity and/or extracellular K^+^ accumulating as a result of neuronal activity initiate and maintain the neurovascular response (Longden et al., 2017). Downstream mechanisms have been proposed to involve recruitment of intermediate cell types, including interneurons and astrocytes, which signal to vascular smooth muscle cells and pericytes (Mishra et al., 2016). Significant experimental evidence suggests that signalling within the neurovascular unit could be mediated by several sequential, parallel, competing and/or redundant pathways involving nitric oxide (NO), prostanoids, purines, amongst others (Attwell et al., 2010; Iadecola, 2017). Hypothesized signalling pathways responsible for the increases in local brain blood flow in response to enhanced neuronal activity are summarized in Fig. 1.

As newcomers to this field we found no up-to-date systematic review of experimental data attempting to analyse the relative significance of the proposed signalling pathways. Addressing this need, we conducted a meta-analysis of publications reporting the results of the experimental studies targeting all hypothesized pathways of neurovascular coupling either pharmacologically or genetically.

**Fig. 2:**
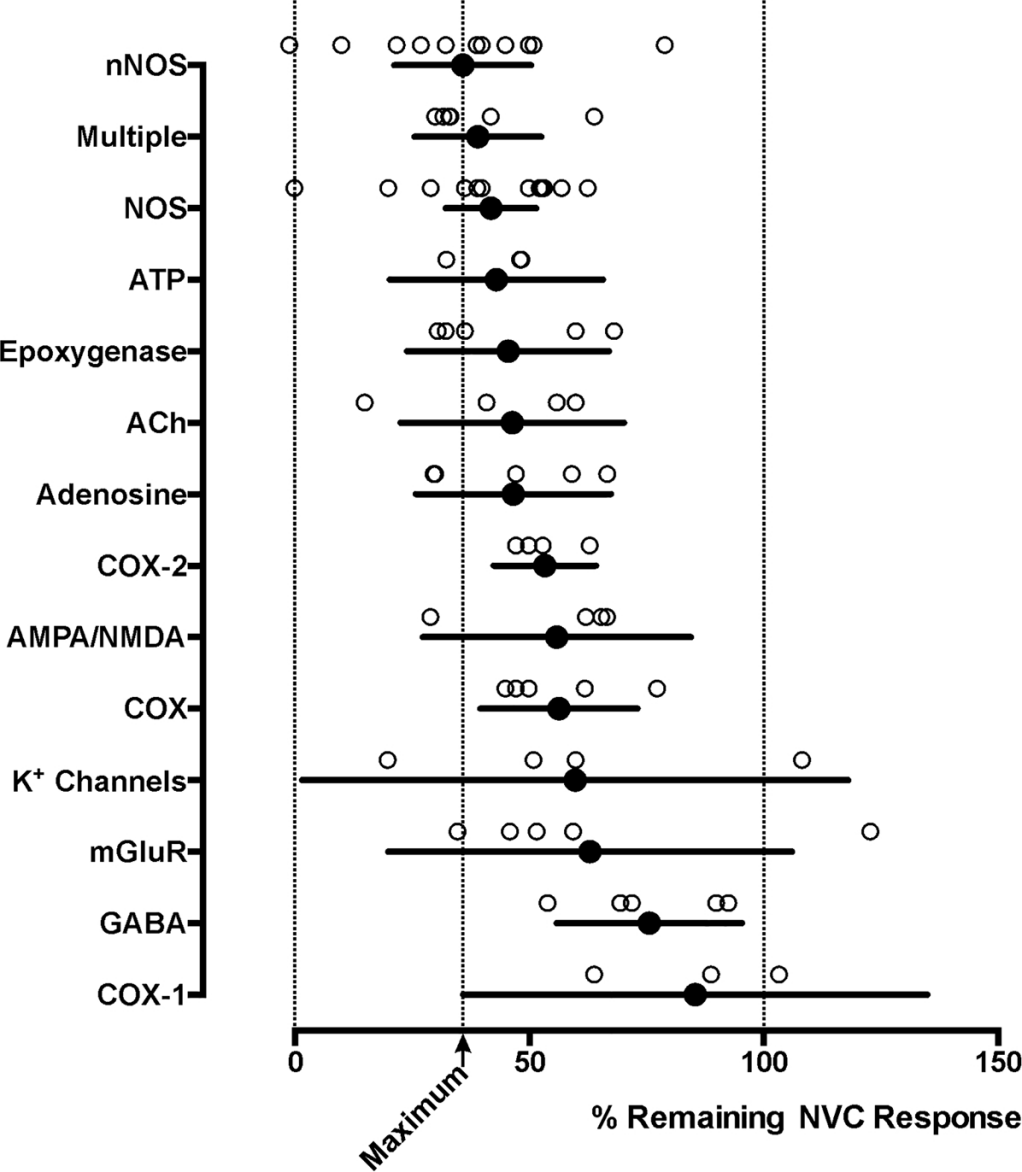
**The effects of blocking hypothesized signalling mechanisms of neurovascular coupling *in vivo.*** Summary plot illustrating the percentage means (with 95% confidence intervals) of neurovascular coupling (NVC) responses that remain in conditions of pharmacological or genetic blockade of hypothesized signalling pathways *in vivo*. Individual data points illustrate the magnitude of the effects of the 79 experimental treatments reported in the publications referenced. *Multiple category includes the results of experimental studies that combined neuronal NOS (nNOS) inhibition with blockade of at least one other target. References: nNOS; (Bonvento et al., 2000; Burke and Buhrle, 2006; Cholet et al., 1997; Iadecola et al., 1993; Kitaura et al., 2007; Lindauer et al., 1999; Offenhauser et al., 2005; Stefanovic et al., 2007; Yang et al., 1999; Yang and Iadecola, 1997; Yang et al., 2003), Multiple; (Dirnagl et al., 1994; Golanov and Reis, 1994; Leithner et al., 2010; Peng et al., 2004; Petzold et al., 2008; Tarantini et al., 2015), ATP; (Mishra et al., 2016; Toth et al., 2015; Wells et al., 2015), NOS; (Adachi et al., 1992; Akgoren et al., 1997; Dirnagl et al., 1993; Golanov and Reis, 1994; Iadecola et al., 1993; Ido et al., 2004; Irikura et al., 1994; Kitaura et al., 2007; Ngai et al., 1995; Peng et al., 2004; Raszkiewicz et al., 1992; Toth et al., 2015; Yang and Iadecola, 1997; Zhang et al., 1995), Epoxygenase; (Lecrux et al., 2011; Peng et al., 2002, 2004), Ach; (Arneric et al., 1987; Biesold et al., 1989; Kocharyan et al., 2008; Zhang et al., 1995), Adenosine; (Dirnagl et al., 1994; Ko et al., 1990; Meno et al., 2005), COX-2; (Bakalova et al., 2002; Lecrux et al., 2011; Niwa et al., 2000), AMPA/NMDA; (Calcinaghi et al., 2011; Lecrux et al., 2011; Petzold et al., 2008; Yang et al., 1999), COX; (Bakalova et al., 2002; Bruhn et al., 2001; Golanov and Reis, 1994; St Lawrence et al., 2003), K^+^ Channels; (Hosford et al., 2018; Longden et al., 2017, 2011), mGluR; (Calcinaghi et al., 2011; Lecrux et al., 2011; Petzold et al., 2008; Sloan et al., 2010; Zonta et al., 2003), GABA; (Kocharyan et al., 2008; Lecrux et al., 2011), COX-1; (Niwa et al., 2001; Petzold et al., 2008).

**Fig. 3:**
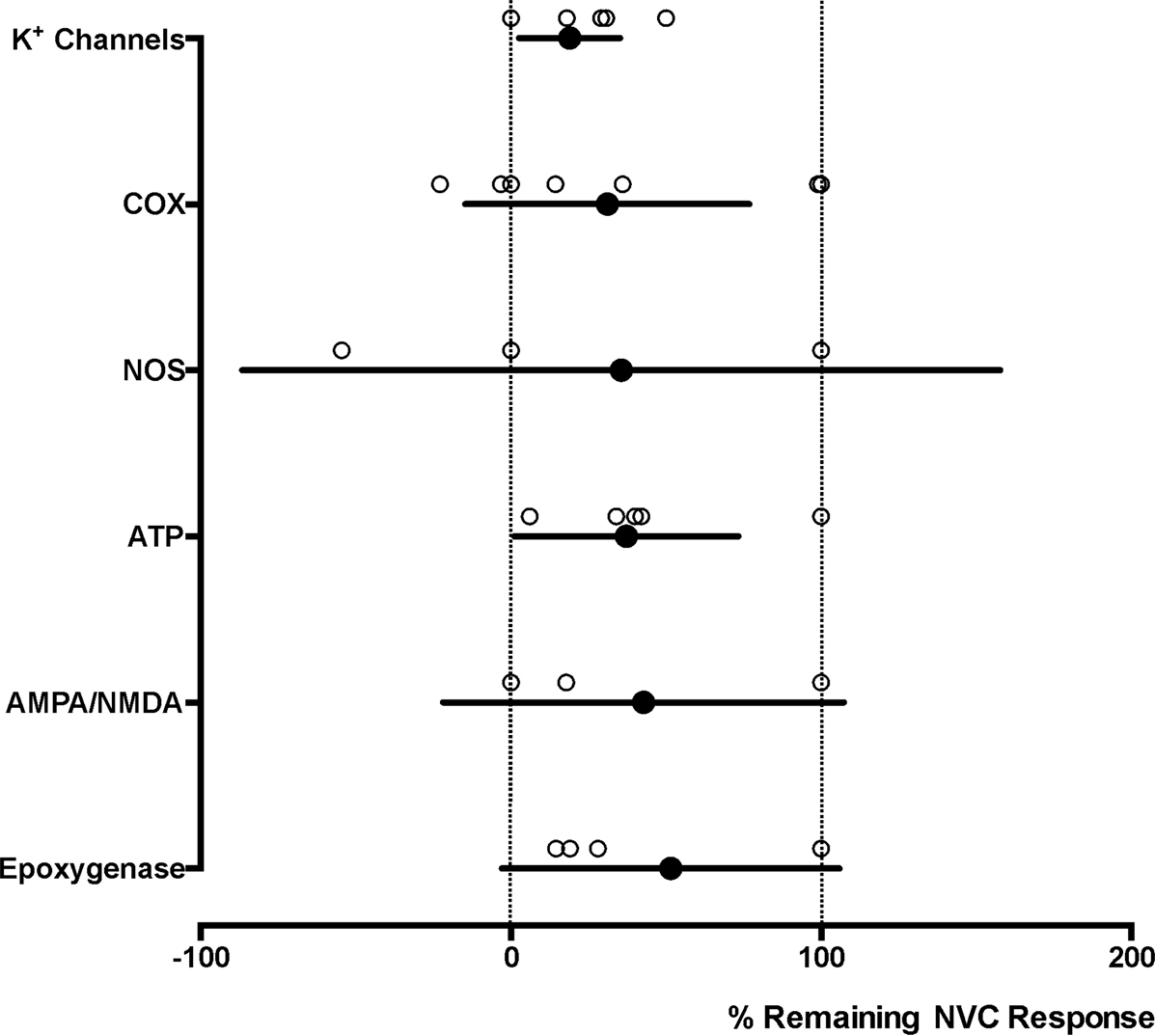
**The effects of blocking hypothesized signalling mechanisms of neurovascular coupling *in vitro*.** Summary plot illustrating the percentage means (with 95% confidence intervals) of neurovascular coupling (NVC) responses that remain in conditions of pharmacological or genetic blockade of hypothesized signalling pathways *in vitro*. Individual data points illustrate the magnitude of the effects of the 36 experimental treatments reported in the publications referenced: K^+^ Channels; (Filosa et al., 2006; Longden et al., 2014, 2017; Longden et al., 2011; Zaritsky et al., 2000), COX; (Filosa et al., 2006; Gordon et al., 2008; Mishra et al., 2016), NOS; (Mapelli et al., 2017; Mishra et al., 2016; Zonta et al., 2003), ATP; (Mishra et al., 2016), AMPA/NMDA; (Hall et al., 2014; Mapelli et al., 2017; Mishra et al., 2016; Zonta et al., 2003), Epoxygenase; (Hall et al., 2014; Mishra et al., 2016).

Initial searches returned hundreds of relevant studies published since the feedforward hypothesis was proposed. In an attempt to distil critical information, meta-analysis was performed using the selection criteria outlined next. Our primary outcome measure was the percent reduction of the cerebrovascular response induced by increased neuronal activity assessed using *in vivo* animal models. The necessary criteria for inclusion were enhanced neuronal activity, recruited by either electrical (*e.g.* forepaw or direct input pathway) or natural (*e.g.* whisker deflection, visual or motor task) stimulation, evoked the neurovascular response measured by recording changes in cerebral vessel diameter, cerebral blood flow, BOLD fMRI or local brain tissue *P*O_2_ in experimental animals. While it is accepted that not all of these measures are exclusively dependent on changes in blood flow only, all have been used as measures of the neurovascular response. Our secondary measure was percent reduction of the cerebral vessel response studied in brain slice preparations *in vitro*. Studies of neurovascular responses evoked by application of receptor agonists (*e.g.* (Busija et al., 2007)), high K^+^ (*e.g.* (Filosa et al., 2006)), direct stimulation of the brain area from which the response was measured or involving pathological models (*e.g.* cortical spreading depression (Ostergaard et al., 2015)) were not included in the analysis. A minimum of two experimental studies *in vivo* targeting the same signalling pathway under the above criteria were required for inclusion. Experimental conditions were grouped into categories targeting hypothesised signalling pathways mediated by NO, prostanoids, ATP, adenosine, K^+^ and those aimed at blocking several targets. Means were taken from the control and experimental group data and the effect of a pharmacological agent or genetic manipulation was expressed as a percentage of the control response. The individual data points were then pooled per targeted signalling pathway and plotted with their respective 95% confidence intervals (Fig. 2, Fig. 3). This analysis standardizes the relative effectiveness of blocking each target or a combination of targets, allowing direct comparisons. Individual data points are tabulated with the original source referenced in Supplementary Table 1 for the primary (*in vivo*) outcome measure.

A systematic review constrained by these criteria returned 61 reports, from which we analysed the results of 79 experimental conditions *in vivo* and 36 experimental conditions *in vitro*. Upon aggregation, blockade of neuronal NOS (nNOS) was found to have the largest effect of blocking any individual target, reducing the magnitude of the neurovascular response by 64% (average of the results of 11 *in vivo* studies) (Fig. 2). Attempts to inhibit multiple targets simultaneously, all of which included nNOS blockade, resulted in a similar reduction of the response magnitude (by 61%; average of the results of 6 studies) (Fig. 2). A potent vasodilator, NO is produced by three NOS isoforms: endothelial NOS (eNOS), nNOS and inducible NOS (Alderton et al., 2001). The temporal profile of inducible NOS activation and deactivation is incompatible with fast control of cerebral blood flow in accordance with neuronal activity. Both eNOS and nNOS are expressed by various cellular components of the neurovascular unit. There is evidence that neurovascular response to somatosensory stimulation is reduced in eNOS deficient mice (Toth et al., 2015). However, other studies reported that blockade of all NOS isoforms with *N*^ω^-nitro-l-arginine (L-NNA) attenuated neurovascular response in both the wild type and eNOS-deficient mice (Ayata et al., 1996) but had no effect in conditions of nNOS deficiency (Ma et al., 1996). Furthermore, the use of non-selective NOS inhibitors was found to have a similar effect on the magnitude of the neurovascular response (reduction by 58%, 14 *in vivo* studies; Fig. 2) to that of a pharmacological or genetic nNOS blockade. It is important to note that NOS blockade is associated with a significant reduction of resting cerebral blood flow (by 28% on average; data retrieved from 6 studies that reported baseline changes). Reduction in basal flow *per se* would be expected to increase the relative magnitude of the evoked neurovascular response (as observed, for example, after caffeine administration (Mulderink et al., 2002)). Depending on the experimental design and particularities of data analysis this may potentially result in overestimation of the neurovascular response in these conditions. However, Iadecola et al. (1993) applied hypocapnia to induce a comparable reduction in cerebral blood flow as observed following systemic NOS blockade and found no effect of this experimental manoeuvre on the magnitude of the neurovascular response in anesthetised rats.

Thus, NO production by nNOS appears to be critically important for the development of the neurovascular response. Yet, a significant proportion (∼1/3) of the response remains after pharmacological blockade of nNOS. This assumes complete blockade, which is likely as genetic nNOS deletion was found to have an effect of similar magnitude as pharmacological intervention (Yang et al., 2003). Almost complete enzyme inhibition is possible with pharmacology: for example, Nitro-l-arginine was reported to inhibit 95 ± 4% of NOS activity when superfused on the cortical surface of anesthetised rats. However, this treatment was associated with a mere 37 ± 4% inhibition of the neurovascular response in the area (Zhang et al., 1995). Another study in anesthetised rats did find a positive correlation between the efficacy of NOS inhibition and the degree reduction of the neurovascular response magnitude in the barrel cortex with  *r^2^* = 0.63 (Irikura et al., 1994). It is important to note that the relative proportion of nNOS expressing neurones varies between different brain areas. In the cerebellar cortex, where the granular layer has the highest level of nNOS expression of all brain areas, blockade of the enzyme was found to completely abolish activity-dependent dilations in slices (Mapelli et al., 2017). In the rodent cerebral cortex, the proportion of nNOS expressing neurones is relatively low (0.5–2%), and all these cells are GABAergic interneurons unevenly distributed between different cortical layers (Valtschanoff et al., 1993). One study conducted in anaesthetized rats, reported that addition of a NO donor can restore the full magnitude of the neurovascular response triggered by whisker simulation in conditions of NOS blockade by superfusion of the brain surface with l-NNA (Lindauer et al., 1999). This suggested that NO might be essential for other signalling pathway(s) to operate by having something what one may call a “permissive” role. One report investigated the effect of systemic NOS blockade with l-NMMA on the neurovascular response in 10 human subjects (White et al., 1999). In contrast to the results obtained in the vast majority of animal studies, NOS blockade had no effect on neurovascular response when the participants performed a finger-tap task.

Several studies targeted another notable hypothesised pathway of neurovascular coupling which involves the release and vasodilator effects (on smooth muscle cells and capillary pericytes) of certain products of arachidonic acid metabolism including prostaglandins (PGs) and epoxyeicosatrienoic acids (EETs). All the enzymes required for the Ca^2+^-dependent production of arachidonic acid and PGs from membrane phospholipids are expressed in astrocytes (Mishra et al., 2016), cells which are often placed at the centre of the neurovascular unit (Fig. 1). PGs (PGE_2_ and PGI_2_ in particular) are potent vasodilators. Recent evidence has implicated PGE_2_ as the dominant prostaglandin mediating neurovascular coupling in the rodent cerebral cortex (Lacroix et al., 2015). However, there is also evidence obtained *in vitro* that PGE_2_ may, under certain conditions, constrict (rather than dilate) brain arterioles of rats and mice (Dabertrand et al., 2013). Systematic review (Fig. 2) has revealed that interfering with this pathway by either genetic deletion or pharmacological blockade of epoxygenase, inhibition of cyclooxygenases (COX) with non-specific agents or blockade of COX-2 specifically, results in a 55, 44 or 47% reduction in the magnitude of the neurovascular response, respectively. In humans, systemic non-specific COX inhibition was also reported to reduce the magnitude of the BOLD response induced by visual stimulation by 47% (Bruhn et al., 2001). It is important to note that indomethacin, used in all these studies as a non-specific COX inhibitor, is also a potent inhibitor of cyclic AMP-dependant protein kinase activity (Goueli and Ahmed, 1980), which is integral to any vasomotor response. Therefore, conclusions drawn from the results of the experiments using indomethacin as a COX inhibitor may somewhat overestimate the importance of this pathway. For example, in humans indomethacin was found to reduce cerebrovascular CO_2_ reactivity, while the other non-specific COX inhibitors (naproxen, ketorolac) had no effect on CO_2_-induced dilations (Hoiland et al., 2016). On the other hand, the effects of specific inhibition or genetic elimination of COX-2 on neurovascular response were found to be similar to that of indomethacin, suggesting that off-target activity of this drug may not be relevant in this context. Specific pharmacological or genetic blockade of COX-1 activity was reported to reduce the neurovascular response by 24% on aggregate (Fig. 2). Based on this analysis it would be logical to suggest that the actions of PGs and EETs mediate the proportion of the neurovascular response which remains after nNOS blockade. However, combined blockade of NOS and COX or NOS, COX and epoxygenase activity reduces the magnitude of the neurovascular response on average by 68% (Golanov and Reis, 1994; Leithner et al., 2010; Tarantini et al., 2015). This falls within the range of the effects observed following inhibition of nNOS activity alone (Fig. 2).

NO, PGE_2_ and EETs dilate cerebral blood vessels by their actions on the arterial smooth muscle cells and/or capillary pericytes, and, therefore, act as the “effector” molecules of the neurovascular response. Studies of the mechanisms underlying the *initiation* of the response focused on the signalling molecules released by active neurones. Previously favoured models of the neurovascular coupling mechanism were centred on synaptic glutamate triggering Ca^2+^-dependent release of vasoactive substances by astrocytes (Attwell et al., 2010). However, this model has been challenged by the evidence showing that mature astrocytes may not express appropriate glutamate receptors (Sun et al., 2013; but also see Nizar et al., 2013) and that neuronal activity-induced changes in flow are not affected in the absence of astroglial IP_3_ receptors (Jego et al., 2014; Nizar et al., 2013; Takata et al., 2013). We proposed in 2015 that (instead of glutamate) the release of purine nucleotide ATP may mediate signalling between neurones and astrocytes (Wells et al., 2015), a conclusion supported by the results of another study involving our laboratory (Mishra et al., 2016). Astroglial IP_3_ receptors would not be essential for the operation of this mechanism as ATP may trigger Ca^2+^ responses in astrocytes *via* activation of ionotropic (P2X) receptors (Mishra et al., 2016). Blockade of ATP actions (by facilitated enzymatic degradation) or application of purinoceptor antagonists was found to reduce the magnitude of the neurovascular response by 57% on average (Fig. 2). Neurovascular response was also reported to be affected following blockade of other key neurotransmitter signalling pathways, including AMPA and NMDA receptors (inhibition by 44%), metabotropic glutamate receptor 5 (inhibition by 37%), muscarinic acetylcholine receptors (inhibition by 54%) or destruction of GABAergic neurones/pharmacological blockade of GABA receptors (inhibition by 24%) (Fig. 2).

Released ATP is rapidly broken down to adenosine by the activities of extracellular nucleotidases (Fig. 1). Seven studies targeted adenosine receptors (some are expressed by vascular smooth muscle cells) and reported (on average) a 53% reduction in the magnitude of the neurovascular response (Fig. 2). One study involving combined nNOS and adenosine receptor blockade showed that 42% of the neurovascular response remains in these conditions (Dirnagl et al., 1994), which is in the range of the effects elicited by NOS blockade alone (Fig. 2), indicating no additive effect of adenosine receptor inhibition. In contrast, human studies demonstrated that non-selective adenosine receptor antagonist caffeine increases the magnitude of BOLD response to visual stimulation by as much as 37%, an effect which was suggested to be due to a reduction in the baseline flow (Mulderink et al., 2002). It is important to note that neurones express both the inhibitory (A_1_) and excitatory (*e.g.* A_2A,2B,3_) adenosine receptors, all sensitive to blockade by caffeine, which is also a weak inhibitor of phosphodiesterase activity, important for the maintenance of vascular tone and reactivity (Pelligrino and Wang, 1998).

Another potential player which has been proposed to mediate the neurovascular response is potassium. Original data suggested that Ca^2+^-activated K^+^ (BK) channels on astroglial end-feet release K^+^ which in turn activates inward-rectifying K^+^ channels (K_IR_) expressed by smooth muscle cells, causing hyperpolarisation and relaxation (Filosa et al., 2006). However, deletion of BK channels was found to have no effect on the neurovascular response (Girouard et al., 2010). A modified hypothesis suggested that enhanced neuronal activity increases extracellular K^+^ sufficiently to trigger smooth muscle relaxation (Longden et al., 2017). Knock-out of a specific subunit, K_IR_2.1, was found to reduce the neurovascular response by ∼50% (Longden et al., 2017), while deletion of K_IR_6.1 had no effect (Hosford et al., 2018). On aggregate, attempts to interfere with K^+^-mediated mechanism(s) resulted in 40% inhibition of the neurovascular response (Fig. 2). The effects of these manipulations in combination with nNOS blockade remain to be determined.

While the data obtained using the *in vivo* preparations are in general agreement between the studies (Fig. 2), the results obtained *in vitro* are largely inconsistent and represented by the extremes (Fig. 3). For example, after NOS or COX inhibition the neurovascular response is either unaffected or completely inhibited (Fig. 3). Accepted major caveats of the *in vitro* approach is that the integrity of the neurovascular unit is compromised and the vessels are not studied at optimal physiological tension, - the limitation which can be partially addressed by application of constricting agents (Filosa, 2010). Studies of the vascular responses using the *in vitro* preparations also necessitate electrical stimulation to be applied in a close proximity to the site from which the neurovascular response is being recorded and could result in a pattern of neuronal connections and/or network recruitment that may not accurately reflect the activities of physiological neuronal pathways. This highlights the importance of studying the mechanisms of neurovascular coupling both *in vitro* and in conditions of intact synaptic connectivity, normal myogenic tone and blood flow. In the opinion of the authors of this analysis, *in vivo* animal models remain to be most useful for studies of the neurovascular coupling mechanisms as experiments involving human participants are also constrained by limitations imposed by the use of generally safe pharmacological agents and non-invasive methods of assessing changes in brain blood flow.

## 4 Regional segmentation

There is emerging evidence of functional regional segmentation of the cerebrovascular tree. Using vascular single-cell transcriptomics Vanlandewijck et al. (2018) demonstrated a clear gradual phenotypic change (zonation) along the continuum of the arteriovenous network in the mouse brain. Recent work has also suggested that the signalling mechanisms responsible for arteriolar *vs* capillary dilations are distinct and involve the release and actions of NO and PGE_2_, respectively (Mishra et al., 2016). However, the majority of studies included in this analysis targeted specific hypothesised signalling mechanisms of neurovascular coupling without taking into the consideration the potential functional regional segmentation of the cerebral vessels capable of active dilation. In the opinion of the authors, the mechanism which controls the vascular tone at the point of most resistance to flow will play the key role in shaping the neurovascular response. According to the recent data reported by Charpak’s group (Rungta et al., 2018) this point (which the authors called “primary functional unit”) could be around the first branching of the penetrating arteriole, as it dilates first in response to increases in local neuronal activity (in the mouse olfactory bulb).

We also do not discount the possibility that the mechanisms of the neurovascular response could vary by the brain region. This analysis included studies of neurovascular coupling in several brain regions, including olfactory bulb, cerebellum, visual, barrel and somatosensory cortical regions with the majority of the experiments carried out in the latter two. Only two studies included in the analysis reported complete abolition of the neurovascular response *in vivo*. In anesthetised rats, the neurovascular responses were blocked in the somatosensory forepaw region of the cortex after nNOS inhibition (Stefanovic et al., 2007) and in the visual cortex, also in conditions of non-specific NOS inhibition (Ido et al., 2004). There is no *in vivo* evidence that the neurovascular response has been effectively blocked in any other brain region. In the absence of a clear consensus on key cell types and vascular segments mediating the neurovascular response in different brain regions, results of all the relevant studies were considered together, pooled and analysed by the targeted signalling pathway.

## 5 Conclusions

This analysis points to the existence of as yet an unidentified mechanism (or mechanisms) of neurovascular coupling which accounts for approximately a third of the neurovascular response. There are other precedents in the history of physiology illustrating the fact that the signalling mechanisms underlying activity-dependent changes in blood flow are sometimes difficult to identify. For example, the nature of the mediator(s) responsible for exercise-induced coronary vasodilation and increases in coronary blood flow (critically important to support myocardial oxygen demands) remains unclear despite similar research interest and significant effort. The involvement of common pathways was experimentally tested, but prostaglandins and K^+^ were found to play no role, while studies involving simultaneous blockade of K_ATP_ channels, NOS, and adenosine receptors demonstrated no effect of the combined treatment on exercise-induced changes in coronary blood flow (Goodwill et al., 2017). On the other hand, if functional hyperaemia is mediated by a single signalling molecule, then the underlying mechanism(s) can be rapidly and unequivocally identified (Burnett et al., 1992).

Considering the importance of maintaining brain energy balance it is likely that there are several parallel signalling pathways evolved to work in concert to shape the neurovascular response and in conditions when one pathway is blocked experimentally, recruitment of the other pathway(s) compensates. However, this raises an obvious question of how the alternative pathways are engaged to maintain (at least partially) the vascular response when the targeted pathway is no longer functional. Some form of a feedback mechanism which can recruit these redundant pathway(s) when the need arises would be required. Alternatively, if the effects of parallel pathways are not simply additive, then a feedback mechanism may not be needed. It is not inconceivable that some form of negative synergy limits the effects of one signalling pathway when it operates in conjunction with the others in the intact system. Finally, in the opinion of the authors, full understanding of the complex mechanisms underlying the development and maintenance of the neurovascular response may emerge if some of the original ideas of metabolic feedback hypothesis proposed by Roy and Sherrington (1890) are re-examined using contemporary experimental approaches.

## Disclosure

The authors declare that there is no conflict of interest.
