# Supplementary material for: What is the key mediator of the neurovascular coupling response?
Source: Neurosci Biobehav Rev. 2019 Jan;96:174–81. doi: 10.1016/j.neubiorev.2018.11.011 (PMC6331662; doi:10.1016/j.neubiorev.2018.11.011)
Supplement: Supplementary file 1 [file mmc1.pdf]

Target	% Remaining NVR	Reference		Target	% Remaining NVR	Reference
**nNOS**	32.3	Bonvento, G., N. et al., 2000		**ACh**	41.0	Arneric, S.P., et al., 1987
(Mean 35.8%)	21.8	Burke, M. and C. Buhrle., 2006		(Mean 46.4%)	60.0	Biesold, D., et al., 1989
	79.0	Cholet, N., et al., 1997			15.0	Kocharyan, A., et al., 2008
	45.0	Iadecola, C., G., et al., 1993			60.0	Kocharyan, A., et al., 2008
	50.0	Kitaura, H., et al., 2007			56.0	Zhang, F., et al., 1995
	38.9	Lindauer, U., et al., 1999		**Adenosine**	59.2	Dirnagl, U., et al., 1994
	40.0	Offenhauser, N., K., et al., 2005		(Mean 46.6%)	66.8	Ko, K. R., et al., 1990
	-1.1	Stefanovic, B., et al., 2007			47.3	Ko, K. R., et al., 1990
	51.0	Yang, G. and Iadecola, C., 1997			29.7	Meno, J.R., et al., 2005
	10.0	Yang, G., et al., 1999			30.0	Meno, J.R., et al., 2005
	27.0	Yang, G., et al., 2003		**COX-2**	50.0	Bakalova, R., T. et al., 2002
**Multiple**	41.9	Dirnagl, U., et al., 1994		(Mean 53.3%)	47.3	Lecrux, C., et al., 2011
(Mean 39.0%)	30.0	Golanov, E.V., Reis, D.J., 1994			53.0	Niwa, K., et al., 2000
	33.3	Leithner, C., et al., 2010			63.0	Niwa, K., et al., 2000
	31.8	Peng, X., C., et al., 2004		**AMPA/NMDA**	66.7	Calcinaghi, N., et al., 2011
	64.0	Petzold, G.C., et al., 2008		(Mean 55.8%)	62.2	Lecrux, C., et al., 2011
	33.3	Tarantini, S., et al., 2015			65.5	Petzold, G. C., et al., 2008
**NOS**	40.0	Adachi, T., et al., 1992			29.0	Yang, G., et al., 1999
(Mean 41.8%)	40.0	Akgoren, N., et al., 1997		**COX**	45.0	Bakalova, R. T., et al. 2002
	62.6	Dirnagl, U., et al., 1993		(Mean 56.3%)	47.2	Bruhn, H., et al., 2001
	39.0	Golanov, E.V., Reis, D.J., 1994			62.0	Golanov, E.V., Reis, D.J., 1994
	20.0	Iadecola, C., G., et al., 1993			50.0	St Lawrence, K.S., et al. 2003
	0.0	Ido, Y., et al., 2004			77.4	St Lawrence, K.S., et al. 2003
	53.3	Irikura, K., et al., 1994		**K+ Channels**	108.3	Hosford, P.S., et al., 2018
	50.0	Kitaura, H., et al., 2007		(Mean 59.8%)	60.0	Longden, T.A., et al., 2011
	52.3	Ngai, A.C., et al., 1995			19.9	Longden, T.A., et al., 2017
	36.4	Peng, X., C., et al., 2004			51.0	Longden, T.A., et al., 2017
	29.1	Raszkiewicz, et al., 1992		**mGluR**	122.9	Calcinaghi, N., et al., 2011
	52.9	Toth, P., et al., 2015		(Mean 63.0%)	59.5	Lecrux, C., et al., 2011
	53.0	Yang, G. and C. Iadecola., 1997			46.0	Petzold, G. C., et al., 2008
	57.0	Zhang, F., et al., 1995			51.7	Sloan, H.L., et al., 2010
**ATP**	32.4	Mishra, A., et al., 2016			34.8	Zonta, M., et al., 2003
(Mean 43.0%)	48.4	Toth, P., et al., 2015		**GABA**	54.0	Kocharyan, A., P., et al., 2008
	48.1	Wells, J.A., et al., 2015		(Mean 75.6%)	72.0	Kocharyan, A., P., et al., 2008
**Epoxygenase**	60.0	Lecrux, C., et al., 2011			90.0	Kocharyan, A., P., et al., 2008
(Mean 45.5%)	30.6	Peng, X, et al., 2002			69.6	Lecrux, C., et al., 2011
	32.3	Peng, X, et al., 2002			92.6	Lecrux, C., et al., 2011
	68.2	Peng, X., C., et al., 2004		**COX-1**	103.4	Niwa, K., et al., 2001
	36.4	Peng, X., C., et al., 2004		(Mean 85.5%)	88.9	Niwa, K., et al., 2001
					64.0	Petzold, G. C., et al., 2008

Supplementary Table 1| Individual data points showing the effect of target pharmacological blockade/genetic deletion in vivo expressed in percent of neurovascular response (NVR) remaining (as plotted in Figure 2) with corresponding references to the original sources.
